# Inhibition of long non-coding RNA NEAT1 suppressed the epithelial mesenchymal transition through the miR-204-5p/Six1 axis in asthma

**DOI:** 10.1371/journal.pone.0312020

**Published:** 2024-10-18

**Authors:** Lei Li, Guoju Li, Renzheng Guan, Hui Ma, Quansheng Xing

**Affiliations:** 1 Respiratory & Cardiovascular Pediatrics Department, The Affiliated Hospital of Qingdao University, Qingdao, Shandong, China; 2 Birth Defect Prevention and Control Centre of Qingdao, Qingdao Women and Children’s Hospital, Qingdao University, Qingdao, Shandong, China; The First Affiliated Hospital of Nanjing Medical University, CHINA

## Abstract

**Background:**

Asthma, a prevalent chronic respiratory condition, is characterized by airway remodeling. Long non-coding RNA (lncRNA) NEAT1 has been demonstrated to participate in airway fibrosis. Furthermore, the miR-204-5p/Six1 axis significantly influences epithelial mesenchymal transition (EMT). However, the function of NEAT1/miR-204-5p/Six1 in asthmatic EMT remains unclear.

**Purpose:**

This study intends to elucidate the function of NEAT1/miR-204-5p/Six1 axis in asthmatic EMT.

**Methods:**

TGF-β1 was used to induce the EMT model in BEAS-2B cells. Immunofluorescence and western blot were executed to verify the establishment of the EMT model. NEAT1, miR-204-5p, and Six1 expression levels were evaluated using RT-qPCR. The role of NEAT1 in EMT *in vitro* was explored by CCK8 assays and flow cytometry. The luciferase reporter assay was performed to validate the interaction between NEAT1 and miR-204-5p/Six1.

**Results:**

NEAT1 expression was increased during EMT. Functional experiments showed that the knockdown of NEAT1 suppressed cell proliferation and promoted cell apoptosis *in vitro*. Furthermore, inhibition of NEAT1 decreased the expression of N-cadherin, vimentin, and α-SMA and increased the expression of E-cadherin. Mechanistically, NEAT1 was identified as a sponge for miR-204-5p, and Six1 was found to be a direct target of miR-204-5p.

**Conclusion:**

Down-regulation of NEAT1 reduced the Six1 expression via targeting miR-204-5p to inhibit the process of EMT in asthma. This study may provide new insight to reveal the underlying mechanisms of asthma.

## Introduction

Asthma is a chronic respiratory system sickness defined by swelling and increased mucus in the airways and airway remodeling (AR) [1]. Environmental and occupational exposures to various substances, including industrial compounds, microbes, and allergens, are primary triggers for asthma development [2]. The global health issue affected an estimated 262 million individuals according to self-reported prevalence data [3]. Short-acting β-agonist and inhaled corticosteroids are clinically primarily used medications to alleviate asthma [4]. However, the treatment results are not effective for patients with severe asthma, which will impose a significant burden on individuals and the healthcare system [5].

AR represents a structural alteration in the airway wall, arising from repetitive cycles of injury and subsequent repair, which are driven by chronic inflammation [6]. As a key feature of asthma, it is generally considered an irreversible structural change [7]. Epithelial mesenchymal transition (EMT) is recognized as an initiating factor in asthmatic AR [8, 9]. TGF-β1 is a key driver of EMT, inducing characteristic cellular changes in asthmatic patients [9]. Elevated levels of TGF-β1 have been observed in both the airway epithelium and submucosal layers of asthma patients [10]. This cytokine leads to bronchial smooth muscle hyperreactivity and AR [7]. Therefore, inhibiting the EMT process may be beneficial for relieving asthma.

With the advancement of next-generation sequencing technology, numerous non-coding RNAs (ncRNAs) have been uncovered [11]. Based on the length and structure of ncRNAs, they can be classified into two basic types: housekeeping and regulatory ncRNAs [12]. Long non-coding RNAs (lncRNAs) and microRNAs (miRNAs) belong to the regulatory ncRNAs [13]. LncRNAs are non-coding transcripts of more than 200 nucleotides [14]. The miRNAs consist of around 20–24 nucleotides and interact with mRNA’s 3’ UTRs to regulate post-transcriptional gene suppression [15]. Recent studies have revealed that lncRNAs act as competitive endogenous (ceRNAs) for miRNAs to regulate miRNA activity [16]. LncRNA NEAT1 was reported to participate in the process of fibrosis in asthma [17]. In addition, miR-204-5p inhibited the proliferation and extracellular matrix (ECM) production of airway smooth muscle cells (ASMC) by regulating Six1 in asthma [18]. However, the functions of NEAT1 and miR-204-5p/Six1 in the EMT process remain unclear.

In this study, the EMT model was induced by TGF-β1 *in vitro*, and the expression of NEAT1 was detected in this model. Next, the function of NEAT1 in the EMT process was further explored, and cell proliferation and apoptosis were detected after inhibiting the expression of NEAT1. Furthermore, the role of NEAT1 in the EMT process was further investigated through western blot and RT-qPCR. Additionally, the potential targeting relationship of NEAT1 with miR-204-5p/Six1 was investigated. Conclusively, our study aimed to investigate the impact of NEAT1 on the EMT process and its potential associated with miR-204-5p/Six1.

## Materials and methods

### Cell culture and treatment

The BEAS-2B, human bronchial epithelial cell line, was purchased from X-Y Biotechnology (China), and the mycoplasma infection was not detected. The cells were cultured in Dulbecco’s Modified Eagle Medium (DMEM, NoninBio, C3113-0500, China) with 10% fetal bovine serum (FBS, Gibco, 10270–106, USA) and 1% penicillin-streptomycin (Solarbio, P1400, China) at 37°C with 5% CO_2_. To establish the EMT model, 10 ng/ml TGF-β1 (MCE, HY-P7118, USA) was added to the DMEM, and the cells were incubated for 48 h.

### Western blot

The BEAS-2B cells were the negative control (NC) group, while cells treated with TGF-β1 were the experimental group. The cells were lysed using RIPA lysis buffer containing 1 mM PMSF (Solarbio, R0010, China) and incubated on ice for 25 min. The supernatant was collected by centrifugation at 12,000 rpm for 10 min at 4°C. The BCA kit (Solarbio, PC0020, China) was used to determine the protein concentration. A total of 10 μL protein was separated using 10% SDS-PAGE at 80 V for 35 min, followed by 120 V for 60 min. Next, the proteins were transferred onto PVDF membranes (Millipore, ISEQ00010, USA) and blocked with 5% skimmed milk (Solarbio, D8340, China) for 2 h at a slow speed on the shaker. The membranes were incubated overnight at 4°C with the following primary antibodies: E-cadherin (1:1,000, bs-1519R, Bioss, China), N-cadherin (1:1,000, A19083, Abclonal, China), vimentin (1:1,000, A19607, Abclonal, China), α-SMA (1:1,000, bsm-52396R, Bioss, China), and β-actin (1:10,000, EM21002, Huabio, China). The membranes were washed three times with TBS buffer (Boster, AR0031, USA) containing 0.1% tween-20 (TBST, Solarbio, T8220, China). The antibodies for E-cadherin, N-cadherin, vimentin, and α-SMA were incubated with horseradish peroxidase (HRP)-conjugated goat anti-rabbit IgG (1:10,000, K008, Bioss, China) at room temperature (RT) for 1 h. The β-actin antibody was incubated with HRP-conjugated goat anti-mouse IgG (1:10,000, K009, Bioss, China) at RT for 1 h. Finally, the membranes were developed using ECL reagent (NCM Biotech, P10300, China) for 1 minute, and the protein bands were analyzed using ImageJ software.

### Immunofluorescence

The BEAS-2B cells were the NC group, while cells treated with TGF-β1 were the experimental group. The cells on slides in 12-well plates were fixed with 4% paraformaldehyde (PFA, Bioss, C0-06002, China) for 15 min and then washed three times with TBST for 3 min each time. Next, 0.5% Triton X-100 (Solarbio, T8200, China) was added for 20 min to enhance cell membrane permeability. Blocking was performed using normal goat serum (Boster, AR0009, China) before incubation with primary antibodies. The primary antibodies were the same as those used in the Western blot, except for the β-actin antibody, and were used at the dilution ratio of 1:100. After washed three times with TBST buffer, the 488 goat anti-rabbit IgG (1:200, Bioss, bs-0295G-FITC, China) was added to the slides in the dark for 1 h at RT. DAPI (Solarbio, C0065, China) was used for nuclear staining. The results were observed using an inverted fluorescence microscope (Leica, Germany).

### RNA extraction and RT-qPCR

The BEAS-2B cells were the NC group, while cells treated with TGF-β1 were the experimental group. The Trizol reagent (Invitrogen, 10296028, USA) was used to extract total RNA from the cells. Reverse transcription of RNA into cDNA was performed using the Superscript III reverse transcription kit (Invitrogen, 18080051, USA) with the following steps: pre-transposition at 95°C for 5 min, followed by 40 cycles of denaturation at 95°C for 10 s, annealing at 58°C for 20 s, and extension at 72°C for 20 s. The RT-qPCR experiments were performed with SYBR qPCR Mix (Invitrogen, 4472920, USA) according to the manufacturer’s instructions. The β-actin was used to normalize the results. The primers sequences were as follows: NEAT1-forward primer (F): 5′-AGGAAGCTTGGCAAGGAGAC-3′; NEAT1-reverse primer (R): 5′-CCTCCCAGAGGTCAAGTTCC-3′; miR-204-5p: 5′-TTCCCTTTGTCATCCTATGCCT-3′; Six1-F: 5′-ATGTCGATGCTGCCGTCGTT-3′; Six1-R: 5′-CTCCAGGTTTCCGCCTTGCT-3′, β-actin-F: 5′-TCCTCCTGAGCGCAAGTACTCC-3′; and β-actin-R: 5′-CATACTCCTGCTTGCTGATCCAC-3′.

### Vector construction and stable transfection

Three NEAT1 siRNA vectors were synthesized and cloned by Hippobio (China). The BEAS-2B cells with an empty plasmid were the NC group. The cells were cultured in a 12-well plate at a density of at least 2 × 10^4^ cells per well. The three siRNA vectors were transfected into cells using Lipo2000 (Life Technologies, 11668019, USA) in the serum-free medium. The cells were incubated at 37°C with 5% CO_2_ for 6 h. After incubation, the medium was replaced with the normal growth medium, and the cells were cultured for an additional 48 h. The nucleic acid sequences used were as follows: NEAT1 si-1 sense: GGGACAGACAGGGAGAGAUGTT; NEAT1 si-1 antisense: CAUCUCUCCCUGUCUGUCCCTT; NEAT1 si-2 sense: UGGUAAUGGUGGAGGAAGATT; NEAT1 si-2 antisense: UCUUCCUCCACCAUUACCATT; NEAT1 si-3 sense: UGGCUAGCUCAGGGCUUCAGTT; NEAT1 si-3 antisense: CUGAAGCCCUGAGCUAGCCATT.

### Cell proliferation analysis

This section included four groups: untreated cells (NC group), cells treated with TGF-β1 (TGF-β1 group), cells transfected with empty plasmids and treated with TGF-β1 (si-NC + TGF-β1 group), and cells transfected with siRNA NEAT1 and treated with TGF-β1 (si-NEAT1 + TGF-β1 group). Among them, the NC and si-NC + TGF-β1 groups served as control groups. The cells were planted in the 96-well plate incubating for 12 h. Next, 10 μL CCK-8 solution was added to wells incubating for 48 h., and the absorbance values at 450 nm were measured.

### Flow-cytometric analysis

Similar to the cell proliferation analysis, the NC group and si-NC + TGF-β1 group served as control groups. Cell cycle analysis was performed using the Cell Cycle Analysis Kit (4A Biotech, FXP021, China) according to the manufacturer’s instructions. After treatment with 95% ice-cold ethanol at 4°C for 2 h, 0.4 mL of propidium iodide (PI) was added to the cells. The cells were then incubated at 37°C for 30 min in the dark. Cells at different cell cycle time points were detected by flow cytometry and analyzed by Modfit software. For cell apoptosis, the 10^6^ cells were resuspended in 1 ml 1 x binding buffer. Subsequently, 100 μL cell suspension and 5 μL Annexin V-FITC were added to a 5 ml flow tube and incubated at RT for 5 min in the dark. Then, 400 μL PBS and 10 μL PI (20 μg/ml) were added to the cells. Cell apoptosis was assessed using flow cytometry.

### Luciferase reporter assay

This section included four groups: cells co-transfected mimics NC and NEAT1-WT plasmids (mimic NC + NEAT1-WT group), cells co-transfected with mimics miR-204-5p and NEAT1-WT plasmids (mimics miR-204-5p + NEAT1-WT group), cells co-transfected with mimics NC and NEAT1-mutant (MUT) plasmids (mimic NC + NEAT1-MT group), and cells co-transfected with mimics miR-204-5p and NEAT1-MUT plasmids (mimics miR-204-5p + NEAT1-MT group). Among them, the mimic NC + NEAT1-WT and mimic NC + NEAT1-MT groups served as control groups. The BEAS-2B cells were seeded into 12 well-plated and co-transfected with 2 μL corresponding plasmids using the lipo2000. After 48 h of transfection, the cells were lysed. Then, 20 μL lysis lysate was added to the black label plate. The luciferase activities were analyzed with firefly luciferase reaction solution (Biomed, China). The primers were as follows: NEAT1-WT: 5′-ATTGCATTGAATTTCAAGGGAATTTAGTATGTA-3′; NEAT1-MUT: 5′-ATTGCATTGAATTTCTTCCCTTTTTAGTATGTA-3′; miR-204-5p mimics: 3′-TCCGTATCCTACTGTTTCCCTT-5′; Six1-WT: 5′-TTTTTTTTTTTTAAATGACAAAC-3′; Six1-MUT: 5′-TTTTTTTTTTTTAAAACTGTTTC-3′.

### Statistical analysis

All experiments were repeated three times, and all results are presented as mean ± standard deviation. Pairwise samples were carried out using Student’s t-test, and multiple groups were compared using one-way ANOVA. * p <0.05, ** p <0.01, *** p <0.001, and **** p <0.0001.

## Results

### The EMT model in BEAS-2B cells was successfully established

**Fig 1:**
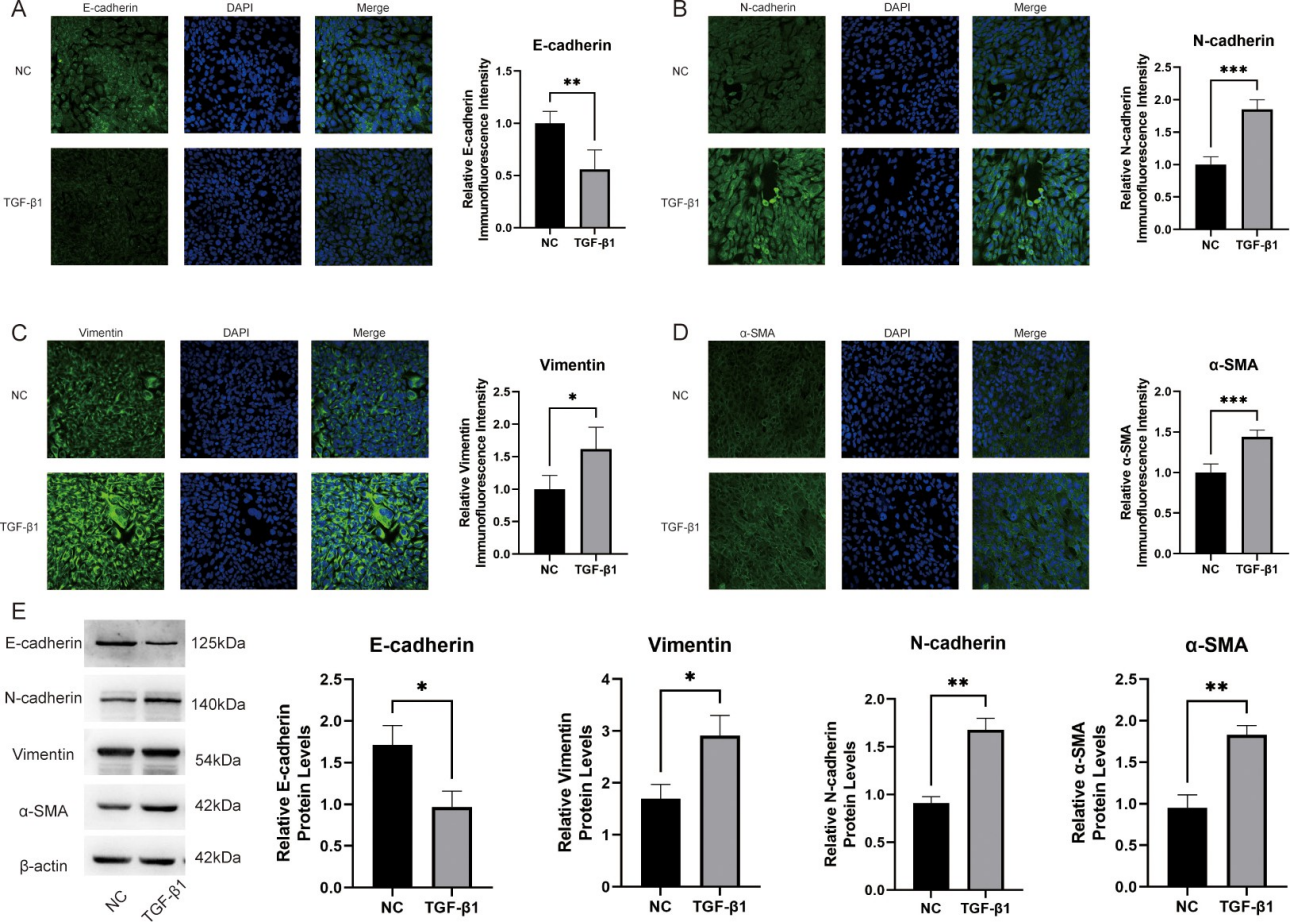
The construction of the EMT model. (A-D) The expression levels of E-cadherin (A), N-cadherin (B), vimentin (C), and α-SMA (D) were determined by immunofluorescence; (E) The expression levels of E‑cadherin, vimentin, N-cadherin, and α‑SMA were detected using Western blot. Experiments were triply replicated for robustness. * p <0.05, ** p < 0.01, and *** p <0.001.

To confirm the successful construction of the EMT model in BEAS-2B cells, immunofluorescence and western blot analyses were employed to assess the expression of the signature protein associated with EMT. EMT is characterized by diminished expression of epithelial cell marker proteins, like E-cadherin, and elevated expression of mesenchymal cell marker proteins, including α-SMA, vimentin, and N-cadherin [19]. Compared with the NC group, the expression of E-cadherin was significantly down-regulated (p <0.01; Fig 1A), while the expression of N-cadherin, vimentin, and α-SMA was significantly up-regulated in the EMT model (p <0.05; Fig 1B–1D). The western blot results were consistent with these findings (p <0.05; Fig 1E). These data indicated that the EMT model was successfully constructed.

### The expression of NEAT1 was increased in the EMT model

**Fig 2:**
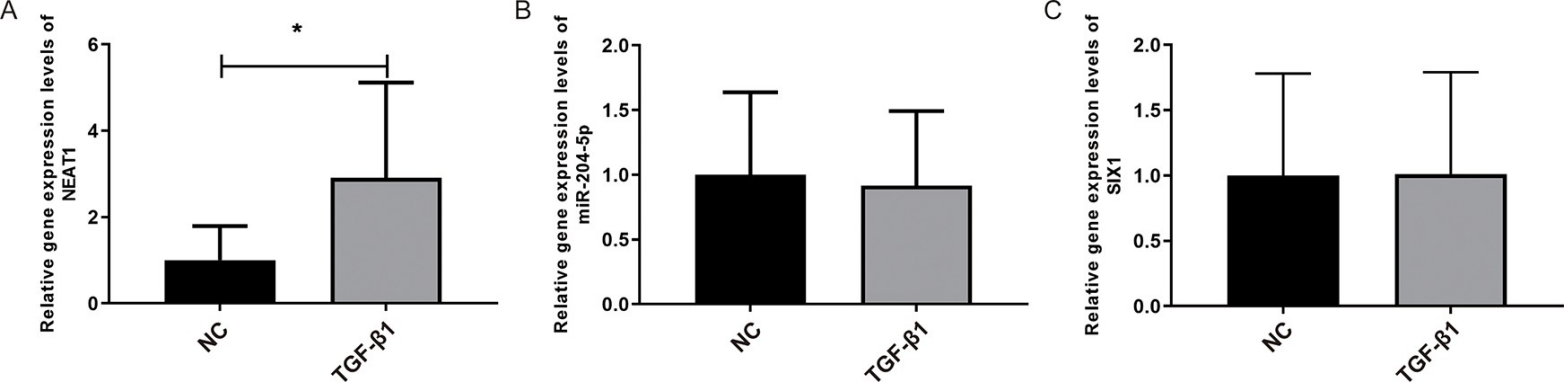
NEAT1 was up-regulated in the TGF-β1-induced EMT model. (A-C) The RT-qPCR analysis shows the expression of NEAT1 (A), miR-204-5p (B), and Six1 (C) in the EMT model. (n = 3). * p <0.05.

We next perform RT-qPCR to detect the expression levels of Six1, NEAT1, and miR-204-5p during EMT. We observed that the NEAT1 expression levels were significantly increased (Fig 2A); however, Six1 and miR-204-5p expression levels did not exhibit significant alterations compared with the NC group (Fig 2B and 2C).

### MiR-204-5p and Six1 exhibited opposing expression patterns in EMT after inhibition of NEAT1 expression

**Fig 3:**
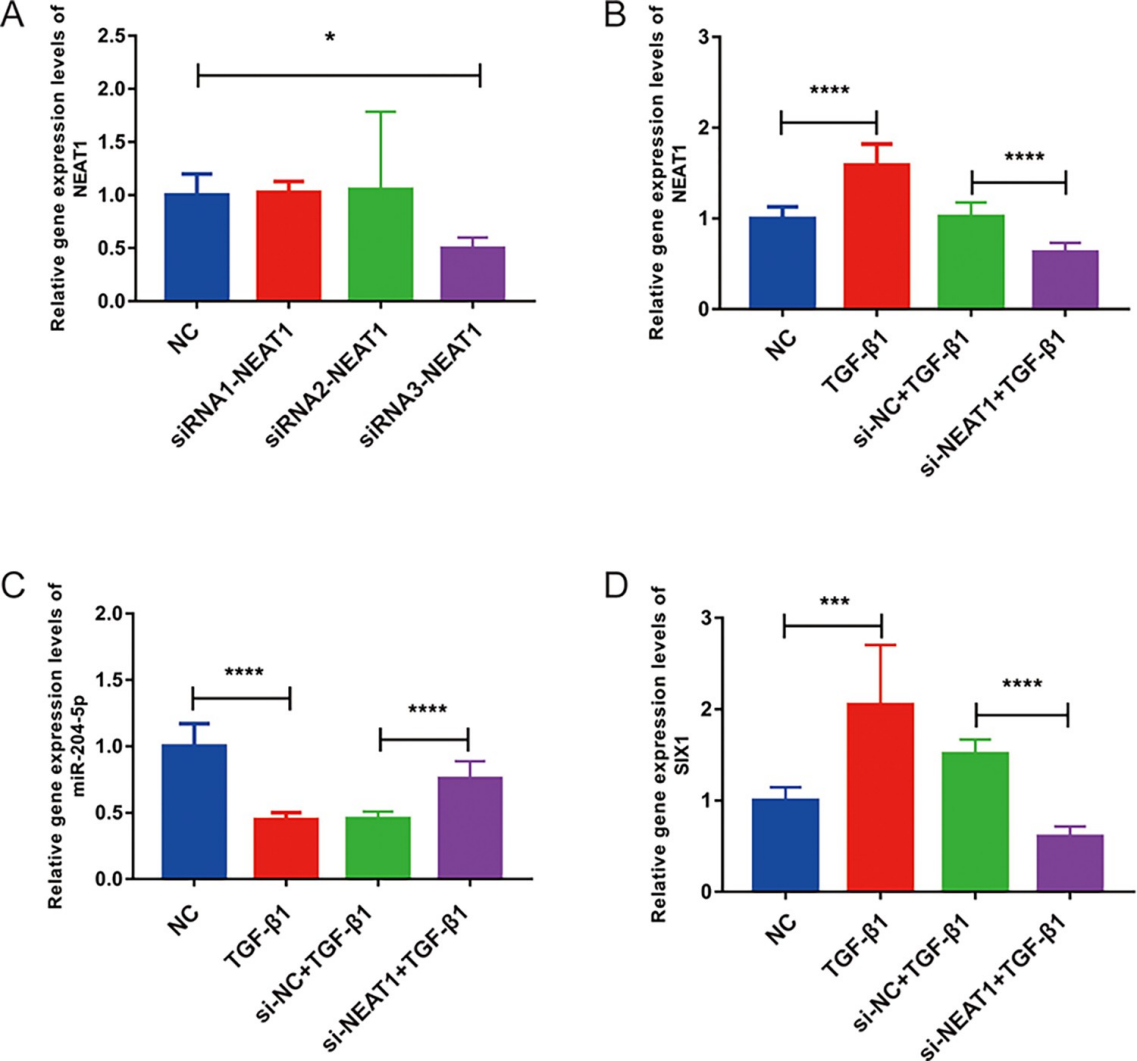
Inhibition of NEAT1 decreased the miR-204-5p expression and increased Six1 expression. (A) NEAT1 expression following siRNA transfection in BEAS-2B cells; (B-D) The expression of NEAT1, miR-204-5p, and Six1 after decreasing NEAT1 expression. * p <0.05, *** p < 0.001, and **** p <0.0001.

To explore the functional role of NEAT1 in EMT, we conducted loss-of-function experiments. We designed and transfected three siRNAs targeting NEAT1 (siRNA1, siRNA2, and siRNA3) along with an empty plasmid into BEAS-2B cells. Within 48 h, the siRNA3 group exhibited a significant reduction in NEAT1 levels compared with the NC group (p <0.05; Fig 3A), indicating that siRNA3 was more effective interference compared to siRNA1 and siRNA2. Consequently, we selected siRNA3 for subsequent experiments. After transfection with siRNA3, NEAT1 expression was significantly decreased in the si-NEAT1 + TGF-β1 group compared with the si-NC + TGF-β1 group (p <0.0001; Fig 3B). MiR-204-5p expression was significantly increased in cells transfected with siRNA3 compared with the si-NC + TGF-β1 group (p <0.0001; Fig 3C). In contrast, Six1 expression was significantly down-regulated in the si-NEAT1 + TGF-β1 group compared with the si-NC + TGF-β1 group (p <0.0001; Fig 3D). The results of RT-qPCR showed that Six1 and miR-204-5p expression could potentially be modulated by NEAT1.

### Silencing NEAT1 suppressed cell proliferation and enhanced apoptosis in the EMT model

**Fig 4:**
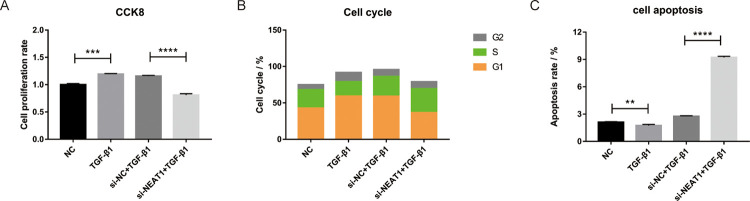
Effect of inhibiting NEAT1 on EMT. (A) Cell proliferation was performed via CCK8 experiment; (B) The percentage of G2, S, and G1 phase cells were detected; (C) The apoptosis of BEAS-2B cells was detected by flow cytometry. ** p <0.01, *** p <0.001, and **** p <0.0001.

To further explore the function of NEAT1 in the process of EMT, siRNA3 was transfected into BEAS-2B cells treated with TGF-β1. CCK-8 assay suggested that the cell proliferation was declined in the si-NEAT1 + TGF-β1 group compared with the si-NC + TGF-β1 group (Fig 4A). Then, the cell cycle and apoptosis were detected by flow cytometry. We observed that downregulation of the NEAT1 resulted in the inhibition of the G1 phase of the cell cycle and increased cell apoptosis in the si-NEAT1 + TGF-β1 group compared with the si-NC + TGF-β1 group (Fig 4B and 4C). All results suggested that the suppression of NEAT1 can impede cell proliferation and enhance cell apoptosis during the EMT process.

### Reducing lncRNA NEAT1 expression may inhibit the process of EMT

**Fig 5:**
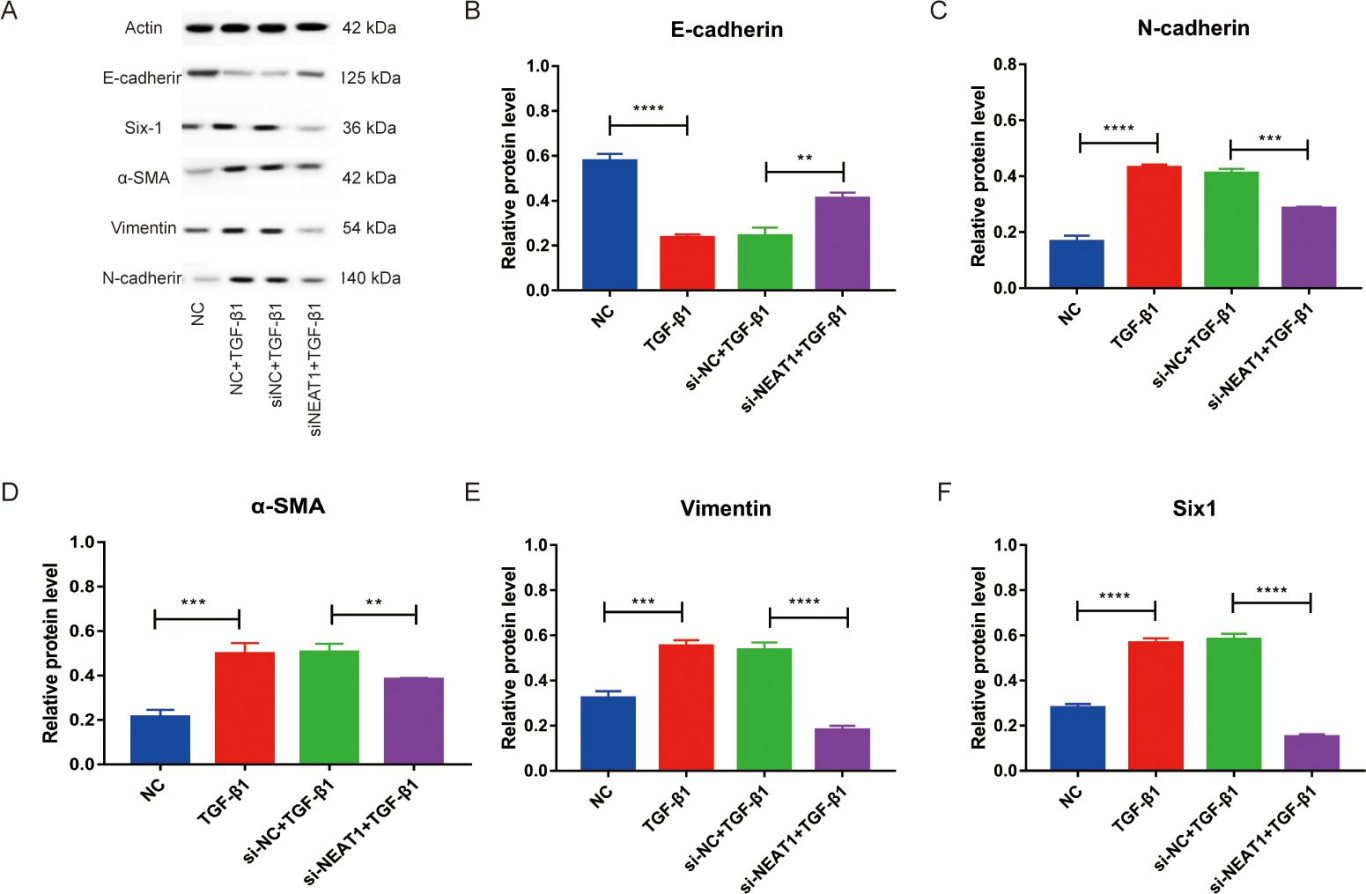
Knockdown NEAT1 suppressed the procession of EMT. (A-F) Western blot was used to analyze the expression of E‑cadherin, N-cadherin, α‑SMA, vimentin, and Six1. ** p <0.01, *** p <0.001, and **** p <0.0001.

Western blot was carried out to explore whether reducing lncRNA NEAT1 expression had an impact on EMT (Fig 5A). When NEAT1 was knocked down, a significant increase in the expression of E-cadherin was observed in the si-NEAT1 + TGF-β1 group compared with the si-NC + TGF-β1 group (Fig 5B). Conversely, the expression of N-cadherin, vimentin, and α-SMA significantly was decreased in the si-NEAT1 + TGF-β1 group compared with the si-NC + TGF-β1 group (Fig 5C–5E). In addition, the Six1 expression was decreased in the si-NEAT1 + TGF-β1 group compared with the si-NC + TGF-β1 group, which is consistent with mRNA levels (Fig 5F).

### NEAT1, Six1, and miR-204-5p have a targeting relationship

**Fig 6:**
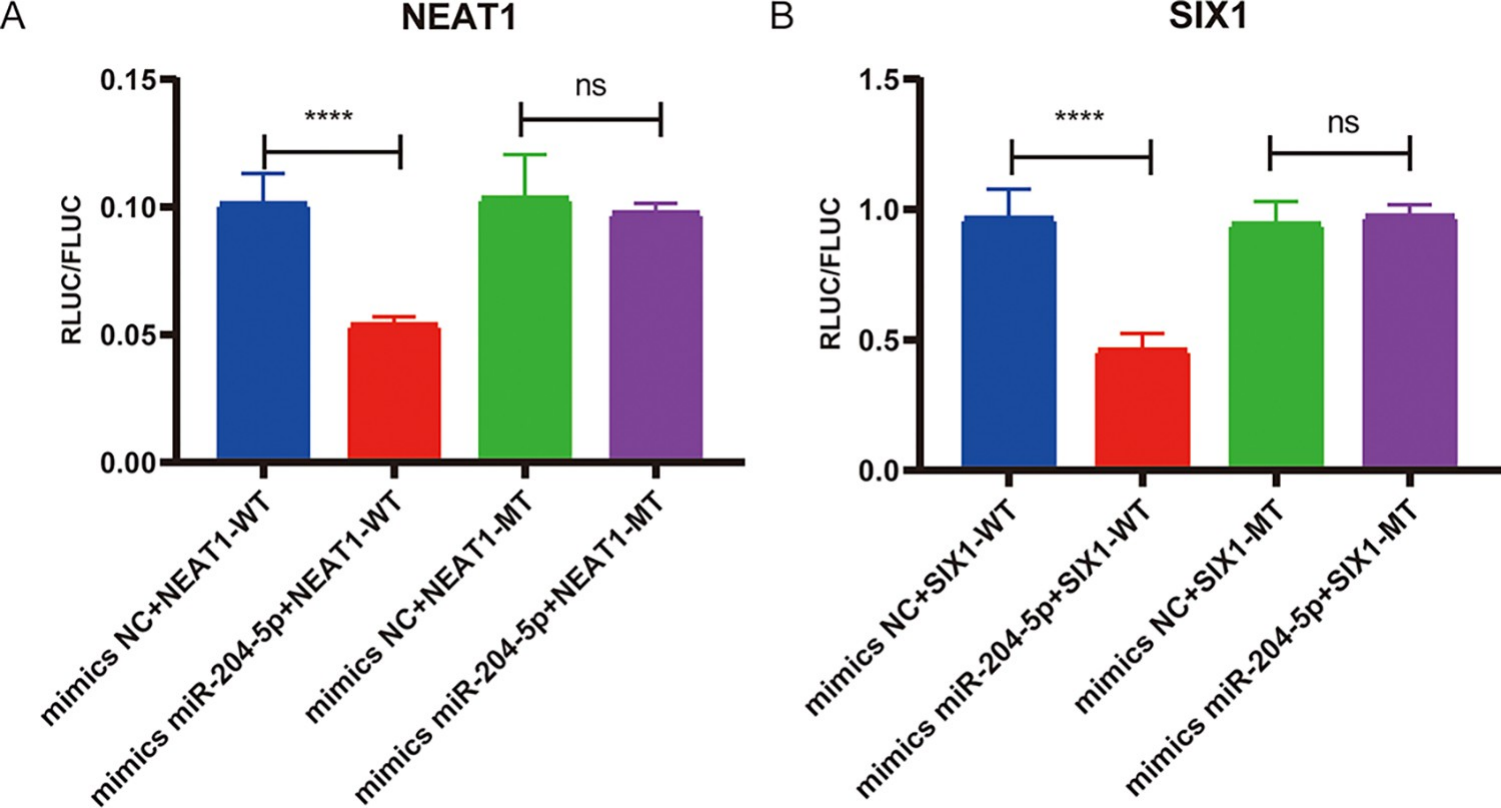
NEAT1 and miR-204/Six1 have a targeting relationship. (A) NEAT1 interacts with the miR-204-5p; (B) Six1 is a target of miR-204p-5p. **** p <0.0001, ns: no significance.

To investigate the potential targeting relationship among NEAT1, Six1, and miR-204-5p, the dual-luciferase reporter assay was carried out. In the mimics miR-204-5p + NEAT1-WT group, the ratio of renilla luciferase activity (RLUC) to firefly luciferase activity (FLUC) was significantly decreased compared with the mimics NC + NEAT1-WT group, while the ratio of RLUC/FLUC was no difference between mimics NC + NEAT1-MT and mimics NEAT1-MT groups (Fig 6A). After co-transfection of Six1-WT and miR-204-5p mimics, the ratio of RLUC/FLUC was significantly reduced compared with mimics NC + Six1-WT group (Fig 6B). The above results indicate that miR-204-5p interacts with the 3’ UTR of NEAT1 and Six1. Taken together, NEAT1 affects the expression of Six1 by competitively binding to miR-204-5p.

## Discussion

In our study, we found that the expression of lncRNA NEAT1 was upregulated in the EMT model. Additionally, inhibition of NEAT1 significantly suppressed cell proliferation and induced cell apoptosis *in vitro*. Moreover, inhibition of NEAT1 could suppress the EMT process. We further demonstrated that NEAT1 positively regulated Six1 expression in the EMT model by competitively combining with miR-204-5p. These results suggested that NEAT1/miR-204-5p/Six1 could be a potential therapeutic target for asthma.

Epithelium damage can induce the EMT process, which exacerbates AR and leads to fixed airflow limitations during asthma attacks [2]. Inhibiting the EMT process can alleviate AR in asthma [20]. LncRNA NEAT1 expression was increased during the EMT process in various diseases, such as cervical cancer, diabetic nephropathy, posterior capsule opacification, and pulmonary fibrosis [21–24]. Recent research has also highlighted the role of NEAT1 in asthma. Li et al. reported that NEAT1 overexpression was associated with asthma exacerbation [36]. Duan et al. also showed that NEAT1 can aggravate inflammation in asthma [25]. In a study involving 170 patients with asthma in exacerbation, 170 patients with asthma in remission, and 170 healthy controls, the expression of NEAT1 was significantly increased in patients with asthma in exacerbation or remission compared with healthy controls [26]. To further investigate the role of NEAT1 in asthma-related EMT, the expression of NEAT1 was examined during the EMT process. We found that the expression of NEAT1 was upregulated during the EMT process, aligning with prior research, indicating its involvement in asthma. After the knockdown of NEAT1, we observed a decrease in cellular proliferation and enhanced apoptosis. These findings align with previous reports in various cell types, including ovarian cancer cells, chondrocytes [27], chondrocyte [28], and lung cancer [29], underscoring the critical role of NEAT1 in cell survival and proliferation.

Recent studies have elucidated the crucial role of EMT in asthmatic AR, which is characterized by airway wall thickening, subepithelial fibrosis, increased smooth muscle mass, and angiogenesis [30]. EMT is an important source of myofibroblasts [31]. In patients with severe asthma, the number of fibroblasts was increased, which can exacerbate airway fibrosis [32]. During EMT, transformed epithelial cells express α-SMA stress fibers and vimentin intermediate filaments, produce ECM components such as collagen and fibronectin, and upregulate matrix metalloproteinases, collectively contributing to AR [33]. Stimulated by cytokines such as TGF-β1, the profibrotic cytokine, fibroblasts can differentiate into myofibroblasts that express α-SMA [34]. These myofibroblasts, in turn, produce more TGF-β1, further accelerating the fibrotic process [34, 35]. The molecular mechanisms of TGF-β1 triggering EMT in the context of AR have been extensively investigated [33]. The TGF-β1/Smad signaling pathway is considered the primary mediator of this process [36]. Xu et al. demonstrated that NEAT1 upregulation could increase α-SMA and vimentin expression, promoting fibrosis and proliferation in the EMT model [37]. In this study, the decrease of NEAT1 up-regulated E-cadherin expression and reduced expression of N-cadherin, vimentin, and α-SMA. These findings suggest that inhibiting NEAT1 may suppress the EMT to alleviate asthma.

Recent studies illustrated that the regulatory roles of lncRNAs/miRNAs/mRNAs in asthma have attracted attention. Yang et al. showed that lncRNA UCA1 might regulate the silicosis-related lung EMT process via the miR-204-5p/ZEB1 pathway [38]. Moreover, miR-106b-5p and miR-203a-3p have been reported to regulate EMT by targeting Six1 [39, 40]. Six1 is a member of the sine oculis homeobox gene family, and overexpression of Six1 induces EMT through up-regulating TGF-β signaling [41]. In asthma mice, the reduction of Six1 expression inhibited TGF-β1-induced EMT [42]. Additionally, the miR-204-5p/Six1 axis has been shown to inhibit ASMC proliferation in asthma [18]. In this study, we investigated the mechanisms underlying NEAT1 and explored whether NEAT1 could regulate the miR-204-5p/Six1 axis. Our findings indicated that the knockdown of NEAT1 significantly increased miR-204-5p expression while increasing Six1 expression during EMT. Moreover, dual-luciferase reporter assays confirmed that NEAT1 promotes the progression of EMT by functioning as a miR-204-5p sponge to modulate Six1 expression. All the above results indicated that the NEAT1/miR-204-5p/Six1 axis might play an important role in EMT.

While our study provides valuable insights into the role of lncRNA NEAT1 in EMT using an *in vitro* model, we acknowledge certain limitations. The NEAT1/miR-204-5p/Six1 axis awaits validation in animal models and asthma-specific contexts. Future research should focus on validating this axis in asthma using animal models and clinical samples, as well as exploring its interactions with other key pathways to enhance our understanding of its role in the development and progression of asthma.

## Conclusion

Taken together, the current results preliminarily suggested that lncRNA NEAT1 played a critical role in EMT via the miR-204-5p/Six1 axis, which provides new therapeutic targets for asthma patients and potentially improves asthma management and patient outcomes. However, *in vivo* experiments were not performed in this study, and future research will validate our findings using clinical samples and animal models.
